# A pedagogical model to enhance nurses' ability to support patient learning: an educational design research study

**DOI:** 10.5116/ijme.62c2.b9c4

**Published:** 2022-07-29

**Authors:** Lena Engqvist Boman, Kay Sundberg, Lena-Marie Petersson, Malin Backman, Charlotte Silén

**Affiliations:** 1 Department of Neurobiology, Care Sciences and Society, Division of Nursing, Karolinska Institutet, Huddinge, Sweden; 2 Department of Learning, Informatics, Management and Ethics, Karolinska Institutet, Stockholm, Sweden

**Keywords:** Educational design research, nurse education, patients' learning, pedagogical model, students' learning

## Abstract

**Objectives:**

To design, apply, evaluate, and analyse a pedagogical model to enhance nurses' ability to create pedagogical encounters to support patients' learning.

**Methods:**

The study relies on an educational design research approach. A pedagogical model based on learning theories was designed, applied, evaluated, and analysed in a specialist nursing programme in cancer care. All students (n=28) who attended the programme accepted to participate in the evaluation of the model. Their perception of the learning activities was evaluated in a questionnaire, and 16 (57%) students responded. The students' learning was assessed in written assignments, including all students. Descriptive statistics, content analysis and theoretical reasoning, were used to analyse data and interpret the usefulness and shortcomings of the model.

**Results:**

The most appreciated learning activities were to study learning theories, observe pedagogical encounters, act as a critical friend, and document one's own pedagogical encounters. The written assignments about observing and performing their own pedagogical encounters with patients showed students' increased awareness of how to support patients' learning. The clinical supervisors' lack of pedagogical knowledge inhibited the feedback on students' performances.

**Conclusions:**

The theoretical analysis of the evaluation identified strengths and needs for further development. The strengths tend to be the ongoing learning process created by learning activities supporting students to continuously study, experience, and apply their knowledge. Nurse supervisors and other stakeholders at the clinics are suggested to be involved in improving the design and require pedagogical competence. Further research should include observational and interview studies related to students' performance in pedagogical encounters.

## Introduction

Patients' knowledge and understanding of their disease and health-related issues have been recognised as prerequisites for participation in treatment and care.^1^^-^^6^ The importance of patient participation for recovery, wellbeing, and patient safety is well documented. Self-management of symptoms and concerns and navigating care are becoming necessary and expected of all patients. Patients with cancer experience the life-altering nature of cancer and treatment outside the clinic and are often left alone to recognise, report, and manage their disease and health recovery.^7^ Preparing nurses with the knowledge and skills necessary to enable patients in effective self-management is imperative. Despite the increasing focus on person-centred health care, the need for information and participation is still unmet.^8^^-^^10^ Initiatives to improve patients' knowledge about their own health often rely on efforts to provide more information. Based on learning theories and research about patients' understanding of and participation in their own care, the importance of recognising patients` learning processes comes to the fore.^1^^-^^4^^, ^^11^^-^^13^ In line with the turn from teacher to student-centred learning in education, a shift from staff directing and giving information to patient-centred learning is needed. To give and provide information to patients is not sufficient to support their understanding. Studies have shown that patients treated for breast cancer are overwhelmed by information, contributing to confusion rather than understanding.^1^^,^^4^ Patients need improved support to process information over time to manage their new situation and participate in their treatment and care. It has been reported in several studies that health care staff lack knowledge regarding the communication process involved in supporting patient understanding and participation.^2^^, ^^4^^, ^^6^^, ^^8^^, ^^14^^, ^^15^ To improve the ability of patients to participate, health care staff, in this case, nurses, need to recognise and pay attention to the meaning of the patients' learning process. Health care staff need to know how to support patients in expressing their understanding and avoiding misunderstandings.^16^ Innovations in curriculum design are called for to support nurses' ability to interact with patients and assess their need for fundamental care.^17^ Today, we lack knowledge regarding how nurse education can be designed to enhance the development of pedagogical competence regarding a shift of focus to patient-centred learning.^2^^, ^^8^^, ^^16^^, ^^18^^-^^20^

The study's theoretical framework is learning theories emphasising that providing the information is only one part of acquiring knowledge and understanding. Learning is regarded as an active, constructive process that requires the information to be processed cognitively, emotionally, and socially through testing and practical actions.^23^ The life-world and pre-understanding of an individual form the basis for understanding, thinking, and action, while learning is constructed through interaction with others and the environment.^24^^-^^26^ Knowles and colleagues^27^ emphasise the importance of considering adults' extensive experience and knowledge, which can be used in learning processes. A basic driving force is a desire to try to understand and manage situations that are perceived as relevant and meaningful.^28^ Understanding is shown in behaviours, thoughts, and attitudes in relation to the outside world.^29^ We would argue that these basic assumptions about learning processes are central to nurses as well as to the patient, although their motives for learning are different. Nurses need to understand how they can learn themselves, as well as how to support patients' learning. Patients are in a vulnerable situation, facing an uncertain future, perhaps with a life-threatening and/or chronic disease and need to understand how to manage self-care and the new situation.^4^^,^^13^^,^^30^

The study relies on the educational design research (EDR) approach,^21^^, ^^22^ applied as a first circuit in an iterative research process. The EDR contains three parts relating to the three core elements - theory, design, and evaluation. The first core element, the theoretical framework, is described above. The second core element describes the design of a pedagogical model based on the theoretical framework and is presented below. The third core element implies the evaluation of the model and is presented in the method and result sections. The results are analysed and discussed in order to improve the model in a second iterative circle of research.

The overall goal of the design of the pedagogical model - the second core element - is to support nurses, as learners, to reach a deeper understanding of what learning might mean for patients and thereby enable them to create meaningful pedagogical encounters. The concept of pedagogical encounters, i.e., learning situations and learning theories enabling an use of ‘think and act learning',^31^ was central to the design of the pedagogical model. A pedagogical encounter is characterised by being a part of a context, a learning environment, and a before, during and after the encounter. The aim of learning is stated in advance and may change depending on what happens during the encounter. All actors involved bring their own intentions and interact with each other in various activities in relation to the current content. Central learning concepts related to 'think and act' learning are pre-understanding, motivation, meaningfulness, learning processes, and metacognition.^31^

The pedagogical model was applied and integrated as a part of an educational programme focusing on the nursing students' own learning, and patients' learning. The application of the model is described below. Details of the model, i.e., the time planning, learning activities, and the content of the model, are presented in Table 1.

As a start, during the first semester students reflect on their previous learning experiences to become aware of their pre-understanding. They study learning theories and discuss them with peers to increase their understanding of learning processes. The concept of a pedagogical encounter is introduced and discussed in seminars with teachers. During the first clinical practice in cancer care, the students are assigned to identify and observe pedagogical encounters and to apply their understanding of learning to patients' situations. To evaluate their ability to process and integrate new knowledge, the students are asked to express their observations, experiences, and reflections on patients' learning as well as their own, in writing and discussions with peers and teachers.

During the second semester, a higher level of application and processing of students' understanding of patients' learning is required. The students plan and perform a pedagogical encounter with a patient while being observed by a 'critical friend' who provides feedback on the performance.^32^ The students also act as critical friends by observing and giving feedback to peers, colleagues, or supervisors. To stimulate reflection, the students are challenged to express their understanding in yet another way. In their written descriptions of the pedagogical encounter, emphasis is placed on the adoption of a meta-perspective on their own learning, and on reasoning based on learning theories, both in general, and regarding patients' learning and understanding.

During the third semester, the students are asked to take the lead by organising and carrying out an interprofessional seminar on patient learning in the clinic. The focus of the learning activity is on the student's ability to facilitate health care staff's learning about patient learning. It places a high demand on the students' understanding and ability to apply what they have learned about pre-understanding, motivation, meaningfulness, learning processes, and metacognition.

**Table 1:** Design of the pedagogical model in semesters 1-3. Descriptions of the aim and learning activities (LA) of the pedagogical model

Semester 1		
Aim: Highlight similarities, differences, preunderstanding, and drivers in individual learning		
	LA	- Interactive lecture about the concept of pedagogical encounters
		- Individual student's previous learning experiences shared with peers
		- Reading and discussions about learning theories with peers and teachers
		- Interactive lecture about reflection as a tool to support the student's own learning
Aim: Identify and deepen understanding of pedagogical concerns for patients in cancer care		
	LA	- Clinical practice 1: Identification and observations of pedagogical encounters between cancer patients and health care staff
		- Individual written assignment about the identification, observation, and analysis of pedagogical encounters in cancer care in clinical practice 1, assessed by the teachers^*^
		- Discussion with peers and teachers about the observed pedagogical encounters
		- Interactive lecture about patient learning
		- Reflections about the individual student's learning and feedback from teachers
Semester 2		
Aim: Deepen the understanding of one's own learning and patient learning		
	LA	- Clinical practice 2: Performance of a pedagogical encounter with a patient in cancer care
		- Practicing the model of 'critical friends' during pedagogical encounters
		- Individual written assignment about the planning, performance, and evaluation of the student's own pedagogical encounter with a patient with cancer in relation to theory and the experience of having and being a 'critical friend' in clinical practice, assessed by the teachers^*^
Semester 3		
Aim: Test one's own understanding of patient learning in an interprofessional context		
	LA	- Interactive lecture about interprofessional learning and planning of a pedagogical encounter involving at least two different professions
		- Planning and leading an interprofessional seminar about how to create a pedagogical encounter for patients relating to cancer prevention and self-care
		- Individual description of the planning, performance, and evaluation of the seminar discussed with a peer, general feedback from teachers in a seminar
		- Final assignment assessing the students' ability to create a cancer prevention intervention for patients with the support of learning theories

^*^The LAs used in the data analysis

The seminars are documented and discussed with peers and teachers. In a final written assignment, the students' understanding of how to support patient learning is assessed. The task is to design a project plan for a nursing intervention, based on theories of learning and communication, regarding the prevention or early detection of cancer.

The objectives of this EDR study are:

    ·   To design and apply the pedagogical model described above, (the first and second core element).

    ·   To evaluate the students' experiences and learning, (the third core element).

    ·   To analyse the evaluation to improve the pedagogical model and illuminate further needs of research, (the third core element).

## Methods

As a third core element in the EDR approach, the application of the design was evaluated.^21^^,^^22^ Educational intervention is complex, and many factors impact the outcome and should therefore be evaluated on different levels. Kirkpatrick^33^ defined four levels of educational outcomes: learner reaction, acquisition of learning, behavioural change, and changes in organisation practice. The present study focuses on the evaluation of the first and second levels, and the research questions are:  How do students taking part in the educational intervention perceive the pedagogical model? How do the students describe and reason about the pedagogical encounters with patients in relation to patients' and their own learning? Collection and analysis of both quantitative and qualitative data were chosen to illuminate answers to the research questions.

### Setting

The educational intervention was carried through in a specialist nursing programme in cancer care at a large medical university in Sweden in 2018. The programme corresponded to 60 credit points and was performed online via a learning platform, including two to three face-to-face meetings per semester and two periods of clinical practice in different areas of cancer care.

### Participants

All students (n=28) who attended the specialist nursing programme in cancer care, in 2018, were invited to participate in the evaluation of the pedagogical model, and all accepted. The students' previous work experience as nurses was considered valuable in encounters with patients. The participants were women with a median age of 36.5 (25–51) years with a median time working as a nurse of 8.5 (1–25) years (n=26, two missing data). Most students worked as nurses while completing the course.

The study protocol was approved by the Stockholm Regional Ethical Review Board. The participants received oral and written information about the study as well as the information on voluntary participation and the option to withdraw at any time without consequences for their studies. Written informed consent was obtained from all participants and the confidential handling of data was guaranteed. Data was analysed by the first and last author who were not involved as examiners in the course.

### Data collection

A study-specific questionnaire was used to collect data related to the first research question concerning the students' perceptions of the learning activities in the pedagogical model. The data collection was done after the students had performed the learning activities in semester 1-2 (Table 1). The questionnaire was distributed in the classroom by the first author to all students who filled in the questionnaire at home and sent it back in a stamped envelope. Sixteen (57%) of the 28 participants responded. The median age of respondents was 39 years (range: 29–51). The median time working as a nurse was nine years (range: 1–23).

All students' individual written assignments related to observing and performing pedagogical encounters with patients were used to gather data related to the second research question about the students' own and patients' learning. The assignments were handed in to the course director, the third author, after completing these learning activities.

#### Questionnaire about students' perceptions of the pedagogical model

The study-specific questionnaire evaluated the students' perceptions of the following learning activities in the pedagogical model: reading learning theories, observing pedagogical encounters, performing a pedagogical encounter, having a critical friend, being a critical friend, writing a reflection log and documenting one's own performed pedagogical encounter. The questionnaire consisted of 26 items, 10 with closed answers and 16 with an open end. Seven items with closed answers were formulated as statements where students could rate to what extent the learning activities supported their learning: i) Not at all, ii) A little, iii) Quite a bit, or iv) Very much. These statements had two follow-up open questions, each for students' comments depending on how they rated the learning activity. For example, "Observing a pedagogical encounter during the clinical practice supported my learning" (rated as above); If you rated the observation of a pedagogical encounter as an activity that supported your learning "Quite a bit" or "Very much", describe what you learned and how; If you rated the observation as an activity that supported your learning "Not at all" or "A little", describe the reason for this and what could have been done differently. Three questions with closed answers concerned where the students performed their pedagogical encounter, who they had been critical friend to and who the student's critical friend had been. Two open-ended questions concerned the students' proposals for development of the pedagogical model and other comments. Two additional questions concerned the students' age and years of work experience as a nurse.

The first and last author developed the questionnaire and for face validity it was discussed with the other authors and revised until consensus was reached about adequacy.

#### Individual written assignments

The first written assignment concerned students' identification, observation, and analysis of pedagogical encounters between health care staff and patients in cancer care during the clinical practice 1 (Suppl. 1 in Appendix). The assignments were assessed by the teachers according to the assessment criteria (Table 2). The second written assignment concerned students' planning, performance, and evaluation of the pedagogical encounters with patients, analysed in relation to learning theory and the experience of having, and acting as, a critical friend in clinical practice 2 (Suppl. 2 in Appendix). The assignments were assessed by the teachers according to the assessment criteria (Table 3).

**Table 2:** Assessment criteria of the first assignment of observing a pedagogical encounter

Describes identified pedagogical encounters	Describes pedagogical encounters in relation to the theoretical model	Describes own learning	Describes patients' learning	Analyses and reflects on observations in relation to learning theory

**Table 3:** Assessment criteria of the second assignment of performing a pedagogical encounter

High quality	Good quality	Poor quality	Fail
Clearly describes the pedagogical encounter and discusses it in relation to the aspects of the theoretical model	Describes the pedagogical encounter to some extent in accordance with the aspects of the theoretical model	Describes the pedagogical encounter poorly and not in relation to the theoretical model	Misunderstands the assignment
Demonstrates an understanding of how patient's learning can be facilitated	Reasons to some extent about how patient's learning can be facilitated	Does not describe how patients' learning can be supported	Does not follow the instructions of the assignment
Demonstrates an understanding of own learning	Does not reason in relation to learning theories	Does not reason in relation to learning theories	
Reasons in relation to learning theories			

### Data analysis

#### Questionnaire about students' perceptions of the pedagogical model

The students' ratings of the statements in the questionnaire were analysed and presented descriptively. Answers to the open-ended questions were analysed on a manifest level according to qualitative content analysis described by Graneheim and Lundman.^34^ Two authors identified categories and, to ensure credibility, they were discussed with the other authors and revised until consensus was reached.

#### Analysis of individual written assignments

The written assignments were analysed using qualitative content analysis.^34^ The basis for the analysis was the instructions for the tasks (Suppl. 1 and 2 in Appendix) and the assessment criteria (Tables 2 and 3). A manifest content analysis was performed to identify and describe characteristics of what and how the students reported from their observations and performances of pedagogical encounters. The quality related to the assessment criteria was analysed and interpreted to capture what students had learned about their own and patients' learning. The assignments were read several times and meaning units related to features of learning were identified. Two of the authors read the assignments and made individual analyses. The authors compared their respectively identified meaning units and discussed their analysis to reach a consensus on how to interpret the students' understanding of patients' learning in relation to their role and knowledge as nurses. The characteristics and quality of students' learning were categorised into four groups: high quality, good quality, poor quality, and fail. All authors contributed with different perspectives regarding the analysis and ensured credibility.

**Figure 1:**
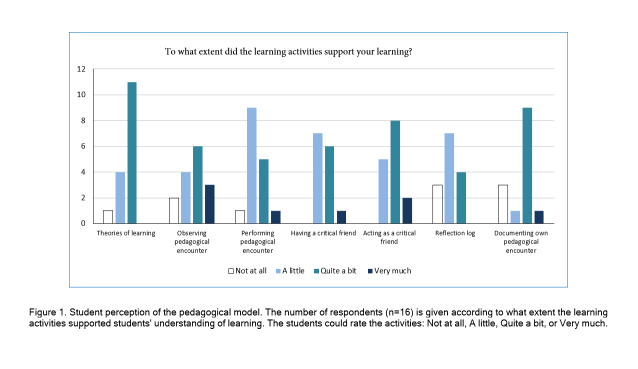
Student perception of the pedagogical model. The number of respondents (n=16) is given according to what extent the learning activities supported students' understanding of learning. The students could rate the activities: Not at all, A little, Quite a bit, or Very much

## Results

### Questionnaire about students' perceptions of the pedagogical model

#### Students' ratings

The most supportive learning activities, as rated by the respondents as "quite a bit" or "very much" were studying learning theories, observing a pedagogical encounter, acting as a critical friend, and documenting one's own pedagogical encounters. The less supportive learning activities were the students' own performance of a pedagogical encounter, having a critical friend, and the reflection log (Figure 1).

The learning theories were most appreciated by those with the most work experience (Md. 14 years). The observations, acting as a critical friend, and documenting one's own pedagogical encounter were rated as supportive by those who had worked a median of 12 years. Those with less work experience as nurses (Md. 8 years) rated the theories, observations, being a critical friend, and documentation of a pedagogical encounter as less supportive for their learning.

#### Students' comments

Analysing the students' comments on their perceptions and reflections related to the different learning activities resulted in three categories: Opportunities to observe and reflect contribute to learning, Learning by one's own performance is conditional and Formulating experiences and relating to theories support learning. The categories are presented below and describe facilitating and hindering factors connected to students' learning.

#### Opportunities to observe and reflect contribute to learning

Observing pedagogical encounters led by other health care staff, i.e., doctors and other nurses, created time and space for considerations. This was the case when students were asked to observe any identified pedagogical encounter on the ward and when they acted as a critical friend to a peer or a nurse colleague. Being an observer, and not occupied by their own responsibility towards the patient, they reflected on the nurses' and doctors' behaviour, what was said, the interactions of all participants, and the patients' reactions. Acting as a critical friend increased attentiveness during the encounter and the awareness of others' performance as well as their own. Good examples of pedagogical encounters offered opportunities to learn, as did poor examples. However, it was difficult to provide feedback to an observed colleague about a pedagogical encounter that was not performed well.

#### Learning by one's own performance is conditional

Several different conditions influence students' perceptions of the possibility to learn through their pedagogical encounters. Having time to plan, perform, and reflect on a pedagogical encounter during clinical practice were expressed by the students as contributing to learning. A factor that limited learning was the feeling of not knowing enough to take responsibility for an unfamiliar diagnosis or treatment, as a nurse in a new environment. Another limiting factor for learning had to do with the opposite perception. Some students perceived a task as too similar to work they did often to contribute to learning. Having a critical friend observing one's performance was a rewarding learning experience if the critical friend was able to contribute with constructive feedback. Feedback and dialogue increased awareness by offering an opportunity to compare thoughts and discuss similarities and differences in views and alternative actions. Feeling safe, having trust in a critical friend, receiving feedback, acknowledging both great performance and areas for improvement, were all important for the learning experience.

#### Formulating experiences and relating to theories support learning

The learning process was supported by linking observation of colleagues and the experience of pedagogical encounters with feedback and through describing the process in writing.

Relating to learning theories, and patients' learning, contributed to an extended view of the pedagogical meaning of an encounter. In cases where the students were able to relate to theories of learning, these contributed to a deeper analysis and understanding of the various factors influencing the encounters. The documentation tasks offered opportunities to structure the experience of pedagogical encounters and to reflect more deeply from a pedagogical standpoint. Applying theories and receiving feedback from teachers increased awareness of various drivers and ways of learning, and contributed to meaningfulness, new insights, and tools.

### Learning features in the individual written assignments

#### Characteristics and quality of assignments about observed pedagogical encounters

All students completed an individual written assignment about the observed pedagogical encounters during their clinical practice 1. Observations were primarily done at surgical and oncological outpatient clinics in cancer care, where patients received their cancer diagnosis and treatment. All but one student (n=27) could identify both planned and unplanned pedagogical encounters between patients and health care professionals, and a median of 3 (0–8) observations per student were reported. In most of the reports, the students described and reflected on patients' learning (n=23), but not on their own learning, which was reported on by only 8 participants. The actors in the pedagogical encounters were mostly doctors, nurses, and patients, with relatives participating occasionally. Some students also observed patients' encounters with other professionals such as physiotherapists, dieticians, and anaesthesiologists. The aim and content of the encounters were to inform about a cancer diagnosis, treatment with chemotherapy or radiotherapy, preoperative preparations, and follow-up after treatment.

The assessment according to the criteria presented in Table 2, revealed variation in the quality of the assignments, i.e., some descriptions included an analysis and reasoning about the observed pedagogical encounters, while others did not. Analysis and reasoning also varied in relation to the use of literature. Most students (n=17) reflected on the aspects of the theoretical model of pedagogical encounter, even if they did not explicitly refer to the literature. A few (n=4) related their analysis briefly to learning theories or their applicability to health care in general. The students' reasoning about the observed pedagogical encounters in relation to the theoretical model also demonstrated their understanding of the learning process through their references to the literature, as exemplified in the excerpt below.

"The interaction during this type of (pedagogical) encounter is extremely important. The patient must have the possibility to tell his/her story and to ask questions about the information he/she receives (the student refers to the literature). It is important that the person who gives the information also controls how it is received. This is especially important when patients receive serious information that may hinder them from thinking and acting." (S 13)

#### Characteristics and quality of assignments about performed pedagogical encounters

All students wrote an individual assignment about their own performance of a pedagogical encounter with a patient and having and acting as a critical friend. Some students performed the pedagogical encounters and the task with the critical friend in their own workplace. The pedagogical encounters were performed at outpatient clinics (n=15), in advanced home care or palliative care (n=10), and in surgical or oncological hospital wards (n=3). In all reported pedagogical encounters, the actors were a patient, the student, and a nurse (colleague, supervisor, peer) with 13 students also reporting that a family member or a friend participated in the encounter. The aim and content were primarily to inform patients about treatments and self-care for the side effects of chemotherapy, radiation, anti-bodies, immunoglobulin, brachytherapy, or blood transfusion. Some encounters centred around providing preoperative and postoperative information, e.g., the self-care of a stoma. The encounters performed in advanced home care or palliative care concerned symptom control, e.g., of pain, nausea, and anxiety, but also the overall life situation including family members.

The written assignments (n=28) were assessed according to the criteria presented in Table 3. Most of the assignments showed high quality (n=12) or good quality (n=10). A few assignments showed poor quality (n=4) and two failed. The "high quality"-assignments demonstrated well-planned encounters and included a clear description and discussion in accordance with the aspects of the pedagogical encounter model. These reports also displayed flexibility and an ability to reflect on the student's learning as well as on the patient's situation, and the conditions for learning. The reflections and reasoning were supported by reference to relevant literature and showed that the students also had gained an increased awareness of learning in their observations of others' pedagogical encounters. As one student stated:

"Now, in retrospect, I have realised how important it has been for me, to develop myself, to observe pedagogical encounters performed by others. I have learnt many good things, during the clinical practice, which I use in my pedagogical encounters in my workplace. This would not have been possible without the observations during the clinical practice." (S 11)

In the "high quality"-assignments, the students also described how they could facilitate the patient's learning during the encounter. A basic factor was to establish a dialogue in a safe environment by starting from the patients' questions, preunderstanding, perceptions, and needs. A pedagogical encounter was an opportunity for learning for all actors. An increased awareness about learning contributed to a new approach in the encounter with the patient, as illustrated below.

"Of course, I have done it before, but you rarely have time for reflection, and it became obvious when you actually were thinking about learning during the encounter. I got a new approach regarding the encounter with the patient when I was actively thinking of it as an opportunity for learning." (S 24)

The patients' understanding was followed up continuously mainly by their questions, replies, and emotions but it was not explicitly described. The students highlighted the need for being well prepared and knowledgeable, to structure the encounter as well as being flexible and attentive to reactions and needs of the patient and relatives. Challenges in supporting patients' learning were to adjust the amount of information, lack of time, the patients' vulnerable situation, and to create a dialogue.

The "good quality"-assignments followed the structure of the pedagogical encounter and included aspects of factors that facilitated patient learning but lacked an explicit evaluation of patients' understanding and a deeper analysis and reasoning in relation to learning theories. In the "poor quality"-assignments, the students did not describe the pedagogical encounter clearly, nor how they supported patients' learning, and did not support their reasoning with reference to learning theories. Those who failed had misunderstood or did not follow the instructions of the assignment. The median years of working as a nurse was 1.5 years among those who failed, compared to 9–11 years among the others.

## Discussion

The pedagogical model in this study focused on students' capability to identify and create pedagogical encounters with patients and to apply 'think and act' learning^31^ in the encounters. The learning theories underpinning the pedagogical model emphasise learners' active participation, the role of pre-understanding, and the importance of experiencing meaningfulness and using all senses to gather information and actively process information to reach understanding.^23^^-^^29^ Based on the analysis of the results, it is concluded that the pedagogical model is a valuable approach to increase nurses' pedagogical competence in cancer care. The theoretical analysis of the evaluation identified strengths and needs for development of the pedagogical model and the continuing educational design research process. The strengths tend to be the ongoing learning process created by different learning activities. They encourage students to continuously study, experience, and apply their knowledge which is in accordance with the learning theories behind the design. The students' written assignments pointed to an increased awareness of the significance of patient learning. Perceptions of how different learning activities contributed to their learning processes varied providing information to consider in development of the design. Below, the results of the evaluation are analysed and discussed in order to improve the design of the pedagogical model as part of the third core element in the EDR process.^21^^,^^22^ The strengths and shortcomings of the model are discussed, elements of achieved pedagogical understanding are identified as well as needs of further research.

Introducing the structure and content of the pedagogical encounter, connecting it to different learning activities, and asking students to determine what a pedagogical encounter might mean in clinical practice, worked well. Analysis of the assignments showed that almost all students could identify and create a pedagogical encounter with patients in cancer care. In the students' reports on the observation and performance of pedagogical encounters, they showed an understanding of how to reason through different pedagogical aspects, and most used the given structure. Constructing learning activities for the students, based on a pedagogical structure, supported recognition of the meaning of patient learning in their observations, as well as using it for planning, performance, and evaluation of pedagogical encounters. However, it was also noticed that there is a risk that a given structure can be used instrumentally and uncritically. There were students who followed the format strictly without showing an ability to adapt and reason about the pedagogical encounter from different points of view, while others used the structure in a flexible manner. The flexibility was demonstrated in the students' ability to adapt and modify the structure and their performance in unexpected situations, and thus satisfy certain learning needs of the patients and relatives the students encountered. Possibly, a deeper understanding of learning processes is a prerequisite for flexibility. Even though the learning activities included reading and discussing learning theories with each other and with the teachers, the assignments showed that some students had difficulties applying theories. Improvements of the design may include support to reflect on the meaning of theories and opportunities for the students to receive feedback from their supervisors when performing pedagogical encounters in clinical practice. The involvement of clinical supervisors in student feedback is perhaps the most important learning support that must be improved in the pedagogical model. Feedback has a great influence on learning, but its effectiveness is dependent on the type of feedback and the way it is given.^35^ Boud and Molloy^36^ point out the importance of opportunities for students to develop the ability to evaluate their own performance and to compare their own self-assessment with the assessment of an observer in a dialogue. The critical friend learning activity was meant to offer opportunities for feedback and allow mutual learning to take place between the observed student and the observer. It worked to some extent, but the evaluation also revealed that the pedagogical competence of clinical supervisors needs to be strengthened. It will require that supervisors become familiar with the concept of pedagogical encounters and learn about feedback. To enhance mutual learning between students and supervisors, the supervisors must be involved in the design and application of the pedagogical model.

The students' awareness of patient learning increased, and it is assumed that the learning activities in the pedagogical model complemented each other. However, conducting some of the learning activities seems to be of particular importance, especially those that offered opportunities for reflective practice. Observing pedagogical encounters in the clinic was valued highly by the participants and culminated in rich descriptions and reflections, both in and on actions.^37^ When not actively engaged themselves, the students could use the time to watch, listen, and reflect. They noticed the importance of patients' preunderstanding and difficulties to grasp all information in relation to overwhelming feelings connected to their cancer diagnosis. This is an important observation as these aspects have a great influence on patients' learning and possibility to participate in their treatment and care of cancer.^1^^,^^4^ The learning activity of acting as a critical friend^32^ was also mentioned as a very valuable experience. This emphasises the importance of activities allowing students to step back, apply their knowledge to recognise learning processes, and reflect on their experiences.

The students with more work experience as nurses showed a greater appreciation for some of the learning activities and performed better in their written assignments than those with less work experience. Novice learners have been found to simplify a new subject and take a conclusive position about themselves or others.^38^ This raises questions about how the design of the pedagogical model can be developed to meet the expectations and needs of students with varying theoretical knowledge and work experience. Since relevance and meaningfulness are significant driving forces for learning,^26^^, ^^27^ it is important to create opportunities for the students to explore their pre-understanding about patient learning before they receive suggestions about how to analyse the concept.

Evaluation of patients' learning constitutes an important part of the pedagogical encounter.^4^^,^^11^^-^^13^ In the students' reports, follow-up of patient learning was mostly described by patients' emotions, reactions, questions, and concerns, but the patients' understanding was rarely explicitly evaluated and described. One reason might be that the students' clinical practice was performed during short periods of time, which reduced opportunities to evaluate patient learning over time. Since learning is a process, patients' understanding of what was said and done during a pedagogical encounter cannot be evaluated simultaneously. Patients need time to assimilate and process a vast amount of information in dialogue with health care staff during a cancer trajectory.^4^ The learning activity focusing on the performance of pedagogical encounters should be developed to further stress follow-up on patients' learning.

Innovations in curriculum design and delivery are important initiatives, which require ongoing monitoring and evaluation.^17^ Important features of EDR are the development of both theoretical insights, and practical solutions involving different stakeholders.^21^ All the authors, who are experienced researchers and teachers within the area, participated in the design of the pedagogical model and acted as teachers. Two of the authors are researchers within medical education, one is the course director, one the course examiner, and one works as a nurse and clinical supervisor. The knowledge and experiences in the research team contributed to different perspectives on the educational design. Their collegial networks provided access to relevant stakeholders. Reflexivity was obtained in the team by critical reflection on the researchers' pre-understanding and its impact on the research questions, methodology and interpretation of data. Furthermore, varied evaluation methods are recommended in EDR to obtain outcomes at different levels in Kirkpatrick's model^33^ and the methods used in this study showed complementary findings on levels 1 and 2. For instance, the learning activity to perform a pedagogical encounter with a patient was not highly valued in the questionnaire. However, in the assignments the students demonstrated their ability and appreciation performing and analysing their pedagogical encounter.

### Limitations

The evaluation is related to the students' perceptions and learning, taking part in the pedagogical model. However, data on student learning build on student reports, not on direct data from studying student performance in clinical practice. This means that the results are to some extent indirect and interpreted by the researchers, and hence a limitation of the study. Another limitation is that only 57% of the students answered the questionnaire about their perceptions of the pedagogical model. This means that students' views, illuminating possible important benefits and shortcomings, are not complete. A strength is that the written assignments were collected from all students.

## Conclusions

The strength of the pedagogical model applied in this study, is the ongoing learning process created by different learning activities, helping students to continuously study, experience, and apply the knowledge thus gathered. The design promotes students' own discovery of what patients' learning means. It is equally important for the students to reflect on patients' learning as well as on their own. An important finding about learning is the significance of opportunities to observe and reflect in the clinic when not being actively engaged themselves in the care. To develop the design, several improvements are needed. Special characteristics of patient learning need to be highlighted further and evaluated as a follow-up after the student's performance of pedagogical encounters. Learning activities that support the students' understanding and ability to apply learning theories should be included. Involvement of clinical supervisors in students' learning activities, offering feedback while students perform pedagogical encounters with patients, is necessary. This will require pedagogical competence and educational activities, not only for nurse supervisors but also for other clinical professionals. To further mature the pedagogical model, stakeholders from the clinic should be involved in improving the design of learning activities.

Further research is needed to enhance theoretical understanding of how education can be designed to increase pedagogical competence concerning patients' learning. An additional evaluation on level three of Kirkpatrick's model,^33^ i.e., behaviour change, could increase the understanding of how to support participants' learning processes and learning. This can be achieved through observation and interview studies in the clinic related to students' performance of pedagogical encounters. Another important area to investigate is the relationship between understanding learning processes and flexibility in the pedagogical encounters with patients, as well as how the clinical learning environment can support students' learning. More research is also needed about how the pedagogical model can mature and be adapted to students' previous knowledge and work experiences, and how it affects students' understanding of patients' learning.

### Practical implications

The pedagogical model was designed and applied in a specialist nursing programme in cancer care. Transfer of the design to other health profession educations appears possible since it is based on general learning theories and research about patient learning. However, the characteristics of each profession and specific context must be considered. Pedagogical encounters bring opportunities for learning for all actors involved and need to be recognised and addressed in health care teams to support patient learning and participation.

A shift in the role of nurses from information providers to facilitators of learning suggests that patient learning should be included in the curricula of nursing education. Pedagogical training is required to develop nurses' pedagogical competence in supporting both patients' and students' learning. A practical implication for development of education is to use the EDR approach. It provides a useful structure for a systematic scholarly work as theory and practice inform each other in continuous development.

### Acknowledgements

We would like to thank all the students who kindly participated in the study and Stefan Binbach Engqvist for language help.

### Conflict of Interest

The authors declare that they have no conflict of interest.

## Supplementary material

Supplementary file 1 Appendix. Individual written assignments

## References

[1] Boman LE, Sandelin K, Wengström Y, Silén C (2018). Patients' participation during treatment and care of breast cancer - a possibility and an imperative.. Eur J Oncol Nurs.

[2] Coulter A, Ellins J (2007). Effectiveness of strategies for informing, educating, and involving patients.. BMJ.

[3] Eldh AC, Ekman I, Ehnfors M (2006). Conditions for patient participation and non-participation in health care.. Nurs Ethics.

[4] Engqvist Boman L, Sandelin K, Wengström Y, Silén C (2017). Patients' learning and understanding during their breast cancer trajectory.. Patient Educ Couns.

[5] Hill S. The knowledgeable patient. Communication and participation in health. A Cochrane handbook. Chichester, West Sussex: Wiley-Blackwell; 2011.

[6] Longtin Y, Sax H, Leape LL, Sheridan SE, Donaldson L, Pittet D (2010). Patient participation: current knowledge and applicability to patient safety.. Mayo Clin Proc.

[7] Howell D, Mayer DK, Fielding R, Eicher M, Verdonck-de Leeuw IM, Johansen C, Soto-Perez-de-Celis E, Foster C, Chan R, Alfano CM, Hudson SV, Jefford M, Lam WWT, Loerzel V, Pravettoni G, Rammant E, Schapira L, Stein KD, Koczwara B (2021). Management of cancer and health after the clinic visit: a call to action for self-management in cancer care.. J Natl Cancer Inst.

[8] Siouta E, Farrell C, Chan EA, Walshe C, Molassiotis A (2019). Communicative constructions of person-centred and non-person-centred caring in nurse-led consultations.. Eur J Oncol Nurs.

[9] Westman B, Kirkpatrick L, Ebrahim F, Henriksson R, Sharp L (2018). Patient-reported experiences on supportive care strategies following the introduction of the first Swedish national cancer strategy and in accordance with the new patient act.. Acta Oncol.

[10] The Swedish Agency for Health and Care Services Analysis. Act without impact. Assessment of the Swedish patient Act 2014-2017. [Cited 3 January 2022]; Available from: https://www.vardanalys.se/in-english/reports/act-without-impact/.

[11] Førland G, Eriksson M, Silèn C, Ringsberg K (2017). Sense of coherence: learning to live with chronic illness through health education.. Health Education Journal.

[12] Førland G, Silèn C, Eriksson M, Ringsberg K (2016). Searching and dealing, confirmation and feeling - participants' approaches to learning in a health education setting.. Health Education Journal.

[13] Kristiansen AM, Svanholm JR, Schjødt I, Mølgaard Jensen K, Silén C, Karlgren K (2017). Patients with heart failure as co-designers of an educational website: implications for medical education.. Int J Med Educ.

[14] Ibrahim F, Sandström P, Björnsson B, Larsson AL, Drott J (2019). 'I want to know why and need to be involved in my own care...': a qualitative interview study with liver, bile duct or pancreatic cancer patients about their experiences with involvement in care.. Support Care Cancer.

[15] Larnebratt A, Fomichov V, Björnsson B, Sandström P, Lindhoff Larsson A, Drott J (2019). Information is the key to successful participation for patients receiving surgery for upper gastrointestinal cancer.. Eur J Cancer Care (Engl).

[16] Karazivan P, Dumez V, Flora L, Pomey MP, Del Grande C, Ghadiri DP, Fernandez N, Jouet E, Las Vergnas O, Lebel P (2015). The patient-as-partner approach in health care: a conceptual framework for a necessary transition.. Acad Med.

[17] Kitson AL, Muntlin Athlin A, Conroy T (2014). Anything but basic: Nursing's challenge in meeting patients' fundamental care needs.. J Nurs Scholarsh.

[18] Arraras JI, Greimel E, Chie WC, Sezer O, Bergenmar M, Costantini A, Young T, Kuljanic K, Velikova G (2011). Information disclosure to cancer patients: EORTC QLQ-INFO25 questionnaire.. Expert Rev Pharmacoecon Outcomes Res.

[19] Joseph-Williams N, Elwyn G, Edwards A (2014). Knowledge is not power for patients: a systematic review and thematic synthesis of patient-reported barriers and facilitators to shared decision making.. Patient Educ Couns.

[20] Tobiano G, Marshall A, Bucknall T, Chaboyer W (2015). Patient participation in nursing care on medical wards: an integrative review.. Int J Nurs Stud.

[21] Chen W, Reeves TC (2020). Twelve tips for conducting educational design research in medical education.. Med Teach.

[22] McKenney S, Reeves TC (2021). Educational design research: portraying, conducting, and enhancing productive scholarship.. Med Educ.

[23] Boud D, Keogh R, Walker D. Reflection: turning experience into learning. London: Kogan Page;1985.

[24] Illeris K. Contemporary theories of learning: learning theorists in their own words. In: Illeris K, editor. A comprehensive understanding of human learning New York: Routledge; 2009.

[25] Merleu-Ponty M. Phenomenology of perception (original title: Phénoménologie de la perception). London: Routledge; 2002.

[26] Marton F, Trigwell K (2000). Variatio est mater studiorum.. Higher Education Research & Development.

[27] Knowles MS, Holton EF, Swanson RA. The adult learner the definitive classic in adult education and human resource development. Amsterdam: Elsevier; 2011.

[28] Dewey J. Democracy and education: an introduction to the philosophy of education. New York: Mac Millan; 1916.

[29] Mann K, Gordon J, MacLeod A (2009). Reflection and reflective practice in health professions education: a systematic review.. Adv Health Sci Educ Theory Pract.

[30] Kneck Å, Fagerberg I, Eriksson LE, Lundman B (2014). Living with diabetes - development of learning patterns over a 3-year period.. Int J Qual Stud Health Well-being.

[31] Silén C. Lärande inom medicin och vårdområdet. Att skapa pedagogiska möten i medicin och vård. (Learning in the medicine and health care field. In: Silén C., Bolander Laksov K. editors. Creating pedagogical encounters in medicine and health care. In Swedish) Lund: Studentlitteratur; 2013.

[32] Dahlgren LO, Eriksson BE, Gyllenhammar H, Korkeila M, Sääf-Rothoff A, Wernerson A, Seeberger A (2006). To be and to have a critical friend in medical teaching.. Med Educ.

[33] Kirkpatrick D. Great ideas revisited: revisiting Kirkpatrick's four-level model. Training & Development. 1996;50(1):54-7.

[34] Graneheim UH, Lundman B (2004). Qualitative content analysis in nursing research: concepts, procedures and measures to achieve trustworthiness.. Nurse Educ Today.

[35] Hattie JT, Timperlay H (2007). The power of feedback.. Review of educational research.

[36] Boud D, Molloy E. Rethinking models of feedback for learning: the challenge of design. Assessment & Evaluation in Higher Education. 2013;38(6):698-712.

[37] Schön D. The reflective practitioner - how professionals think in action. England: Arena, Ashgate Publishing Limited; 1993.

[38] Chick N, Karis T, Kernahan C (2009). Learning from their own learning: how metacognitive and meta-affective reflections enhance learning in race-related courses.. International Journal for the Scholarship of Teaching and Learning.

