# Supplementary material for: A pedagogical model to enhance nurses' ability to support patient learning: an educational design research study
Source: Int J Med Educ. 2022 Jul 29;13:176–86. doi: 10.5116/ijme.62c2.b9c4 (PMC9911139; doi:10.5116/ijme.62c2.b9c4)
Supplement: Supplementary file 1 — Appendix. Individual written assignments [file ijme-13-176-S1.pdf]

Appendix

Individual written assignments

Supplement 1. Instruction to the first assignment of observing pedagogical encounters	
	The aim is to develop your ability to identify pedagogical encounters and assess patients' learning needs regarding their disease, treatment, and care. During your clinical practice 1 you are requested to identify and observe various pedagogical encounters the patients participate in. Make use of the text about pedagogical encounters (Silén 2013) and the discussions during the campus day to create a structure for your observations and questions you want to ask patients. Document your observations and patients' deidentified answers to your questions.
Supplement 2. Instruction to the second assignment of performing a pedagogical encounter	
	The purpose of the task is for you to develop your ability to perform pedagogical encounters and assess patients' learning needs regarding their illness, treatment, and care. It also includes for you to give and receive feedback.
	During your clinical practice 2, you will plan and perform one or more pedagogical encounters with a patient and any relatives. Base your planning on what you have learned about pedagogical encounters, literature studies on learning and your own experiences of identifying and observing pedagogical encounters. The planning should include aspects of the pedagogical encounter before, during and after the encounter.
	During your pedagogical encounter you will be observed by a critical friend – a peer, nurse, or supervisor - who will give feedback on your performance. You will also act as a critical friend and give feedback to a peer, nurse, or supervisor on their performed pedagogical encounter.
	Document your planning, implementation, the feedback you have received and your own reflections and link to relevant literature. Describe both your own and the patient's learning.
